# Supplementary material for: Analysis of the robustness and dynamics of spin-locking preparations for the detection of oscillatory magnetic fields
Source: Sci Rep. 2022 Oct 10;12:16965. doi: 10.1038/s41598-022-21232-1 (PMC9550815; doi:10.1038/s41598-022-21232-1)
Supplement: Supplementary file 1 — Supplementary Information. [file 41598_2022_21232_MOESM1_ESM.pdf]

Analysis of the robustness and dynamics of spin-locking preparations for the detection of oscillatory magnetic fields

Milena Capiglioni[1,*], Federico Turco[1], Roland Wiest[1], Claus Kiefer[1]

[1] Institute for Diagnostic and Interventional Neuroradiology, Support Center for Advanced Neuroimaging (SCAN), University of Bern, Bern, Switzerland.

Supplementary Information

1. Simulator details

The used rotation matrices $\boldsymbol{R}_{\hat{x}}(\alpha)$, $\boldsymbol{R}_{\hat{y}}(\alpha)$, $\boldsymbol{R}_{\hat{z}}(\alpha)$ can be represented by

$$\boldsymbol{R}_{\hat{x}}(\alpha) = \begin{bmatrix} 1 & 0 & 0 \\ 0 & \cos(\alpha) & -\sin(\alpha) \\ 0 & \sin(\alpha) & \cos(\alpha) \end{bmatrix},\ \boldsymbol{R}_{\hat{y}}(\alpha) = \begin{bmatrix} \cos(\alpha) & 0 & \sin(\alpha) \\ 0 & 1 & 0 \\ -\sin(\alpha) & 0 & \cos(\alpha) \end{bmatrix},\ \boldsymbol{R}_{\hat{z}}(\alpha) = \begin{bmatrix} \cos(\alpha) & -\sin(\alpha) & 0 \\ \sin(\alpha) & \cos(\alpha) & 0 \\ 0 & 0 & 1 \end{bmatrix}.$$

Each rotation appears counter-clockwise when the axis about which they occur points toward the observer, and the coordinate system is right-handed.

The relaxation matrices $\boldsymbol{A}$, $\boldsymbol{B}$, $\boldsymbol{A_\rho}$ and $\boldsymbol{B_\rho}$ can be represented by

$$\boldsymbol{A} = \begin{bmatrix} e^{-t/T_2} & 0 & 0 \\ 0 & e^{-t/T_2} & 0 \\ 0 & 0 & e^{-t/T_1} \end{bmatrix}, \boldsymbol{B} = \begin{bmatrix} 0 \\ 0 \\ 1 - e^{-t/T_1} \end{bmatrix}, \boldsymbol{A_\rho} = \begin{bmatrix} e^{-t/T_{2\rho}} & 0 & 0 \\ 0 & e^{-t/T_{1\rho}} & 0 \\ 0 & 0 & e^{-t/T_{2\rho}} \end{bmatrix}, \boldsymbol{B_\rho} = \begin{bmatrix} 0 \\ M_{\infty,\hat{y}}(1 - e^{-\frac{t}{T_{1\rho}}}) \\ 0 \end{bmatrix}.$$

$M_{\infty,\hat{y}}$ is the equilibrium magnetization in the double rotating frame along the spin-lock axis, which we neglect for being much smaller than the generated by the static field B_0. Within the simulator, each rotation step of duration dt will have two relaxation steps of $dt/2$, which means evaluating the four relaxation matrices in $t = dt/2$.
To consider that the target field could oscillate along an arbitrary axis, equation (4) can be generalized as

$$\boldsymbol{M}(\tau) = \boldsymbol{R}_{\hat{z}}(\delta(\tau))\boldsymbol{R}_{\hat{x}}(\zeta(\tau))\boldsymbol{R}_{\hat{y}}\big(-\omega'_{eff}(\tau)dt\big)\boldsymbol{R}_{\hat{x}}(-\zeta(\tau))\boldsymbol{R}_{\hat{z}}(-\delta(\tau))\boldsymbol{M}(\tau - dt).$$

Where δ and ζ represent the angles formed by the effective field direction with respect to $\hat{y}$ in the yx and yz plane respectively. It follows that when the direction of the target field is along $\hat{z}$, δ is 0 and we recover equation (5).

To validate the rotation matrix simulator (RM sim), we compared it with the analytical model presented by Ueda et al. 2018 and the numerical solution of the differential equation presented in this same work using the 4th-order Runge-kutta. The analytical simulator is only valid for a sinusoidal field, so we compared the three simulated signals as a function of the amplitude of the target field. As shown in Supplementary Figure 1. a), both numerical methods approximate well to the Analytical solution. However, the Runge-kutta methods needs a smaller time step (dt < 5E-4 s) to match the accuracy of the RM simulator, as can be seen in panel b). For the same time step dt, the calculation

time of the Runge-kutta method is slightly smaller. However, to match the same percentual error, dt must be lower than the required for the RM simulator, making the effective calculation time bigger.

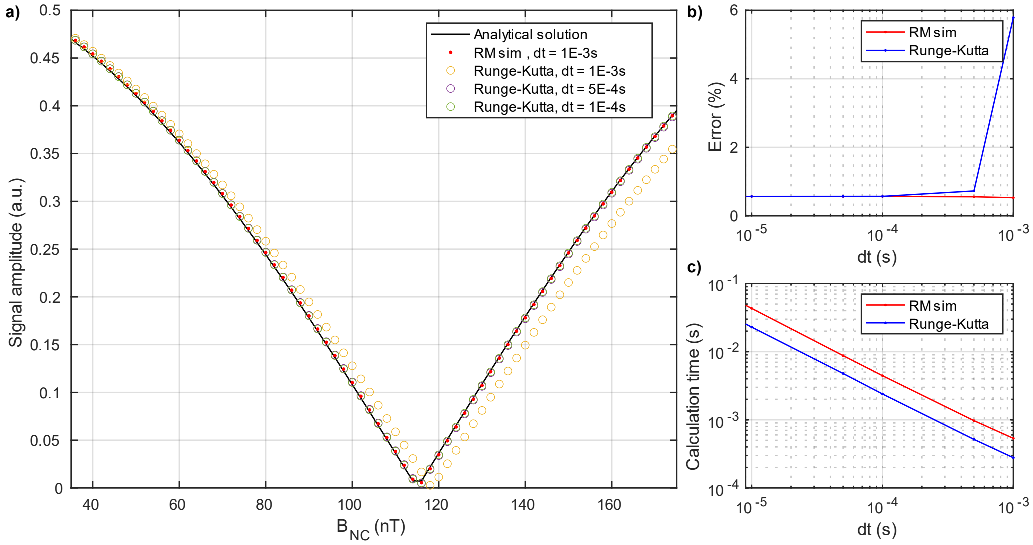

Supplementary figure 1: Validation of the proposed simulation method. a) Signal as a function of the target field amplitude simulated using the analytical solution to the differential equation, the rotation matrix simulator used in this work, and the numerical solution obtained with a 4th order Runge-kutta method. The Runge-kutta was calculated for different times steps until the error (calculated as the mean distance to the analytical solution) was lower than 1%. b) Percentual error as a function of time step for the rotation matrix simulator and 4th order Runge-kutta solution. c) Calculation time as a function of time-step for the proposed rotation matrix simulator and 4th order Runge-kutta solution.

2. Dynamics in the double rotating frame

The double rotating frame is usually employed to depict magnetization dynamics under SL preparation techniques. The double rotating frame (x', y', z') is defined as if the simple rotating frame is now also rotating around the SL axis at the SL frequency. We can decompose the target oscillating field $\boldsymbol{B_{NC}}(t) = B_{NC}\, sin(\omega_{NC}t + \varphi)\hat{z}$ into two fields of half the amplitude oscillating in the xz plane with opposite frequencies as shown in Supplementary Figure 2 i):

$$\boldsymbol{B1_{NC}}(t) = \begin{cases} B_{NC}/2.\, cos(\omega_{NC}t + \varphi)\hat{x} \\ 0 \\ B_{NC}/2.\, sin(\omega_{NC}t + \varphi)\hat{z} \end{cases} \quad and \quad \boldsymbol{B2_{NC}}(t) = \begin{cases} B_{NC}/2.\, cos(-\omega_{NC}t - \varphi + \pi)\hat{x} \\ 0 \\ B_{NC}/2.\, sin(-\omega_{NC}t - \varphi + \pi)\,\hat{z} \end{cases}$$

When in resonance with the SL frequency, one of the fields will be static in the double rotating frame while the other one will be oscillating at $2\omega_{SL}$. Applying the rotating wave approximation (firstly proposed in this context by Ueda et al., Journal of Magnetic Resonance, 2018), we discard the effect that the off-resonance field will have on the magnetization. Supplementary Figure 2 ii), iii) and vi) shows the magnetization dynamics for the three presented SL preparations in the double rotating frame. For BASL there is only one SL frequency, therefore the target field $\boldsymbol{B1_{NC}}(t)$ remains static and deviates the magnetization from the SL axis. For RESL and CRESL, the same happens during the first half of the SL pulse. When the SL changes sign, the opposite field $\boldsymbol{B2_{NC}}(t)$ will be on resonance, and its initial position will depend on the phase accumulated during the first part of the evolution. Depending on this phase, the contrast accumulated during the first SL part can be compensated.

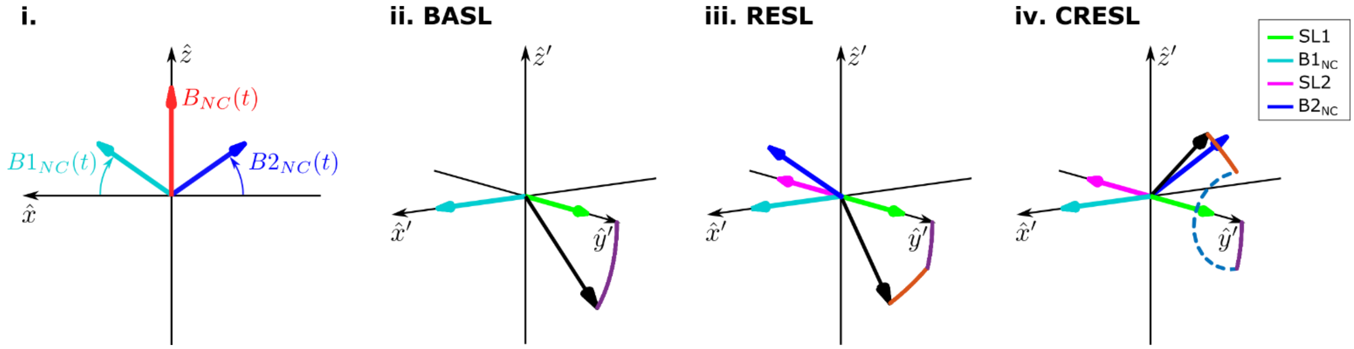

Supplementary figure 2: Magnetization dynamics in the double rotating frame. i) Target field decomposition in the simple rotating frame. ii), iii) and iv) BASL, RESL and CRESL magnetization dynamics represented in the double rotating frame respectively. As the double rotating frame rotates around the SL axis, the sign of rotation changes when SL1 changes to SL2 in the RESL and CRESL cases. Therefore, only $SL1$ and $B1_{NC}$ are present during the first SL part, and $SL2$ and $B2_{NC}$ are present during the second part for RESL and CRESL. The magnetization path follows the same color code as the one used in Figure 1 of the main text. The 180° pulse dynamic is indicated with dashed lines.

3. SL pulse calibration

An error in the excitation angle can be corrected by the change in the sign of the second half of the spin-lock pulse (RESL) and an error in the B_0 field can be corrected by the 180° pulse (CRESL). However, no compensation can be made for the B_1 inhomogeneity influence during the application of the SL pulse. This will induce a F_{SL} different from the one of the target field and the resonance condition will not be fulfilled. To solve this problem, an SL calibration can be made to find the B_1 error for each target frequency. Supplementary Figure 3 a) shows the measurements at different resonant frequencies as a function of F_{SL} and b) the obtained linear regression. The initial phase was set to 0, and 30 repetitions were acquired and averaged for each point on each curve. From the fitted curve, the SL amplitude was corrected before every measurement.

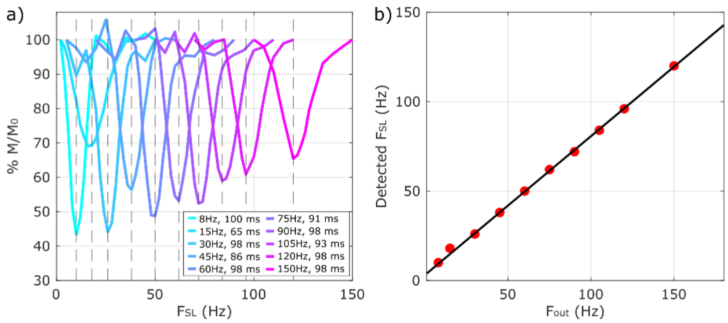

Supplementary figure 3. SL amplitude calibration. a) Percentual change of the signal as a function of F_{SL} for different values of the target oscillating field frequency (F_{out}). b) Linear regression of the detected (F_{SL}) vs the expected frequency (F_{out}). The obtained slope was then used to define the amplitude of each SL pulse.

4. BASL contrast in the presence of field inhomogeneities

To explain the result showed in Figure 3 (b.i) of the main text, we fitted the BASL contrast using the field inhomogeneity as fitting parameter. The excitation angle α, the off-resonance frequency $\Delta\omega_0$ and the spin locking frequency F_{SL} were used as fitting parameters. The rest of the parameters were set as stated in the methods. Supplementary Figure 4 shows the fitting result. The fitted parameters were $\alpha = 73°$, $\Delta\omega_0 = 0$ Hz, and $F_{SL} = 90{,}88$ Hz. This indicates that B_1 is the main contributor to the behavior observed in this case. This behavior comes from the

magnetization without a target field (M_0), when the tip down pulse angle is smaller than 90°. This generates an oscillation around the SL axis that result on a transversal magnetization after the tip up pulse.

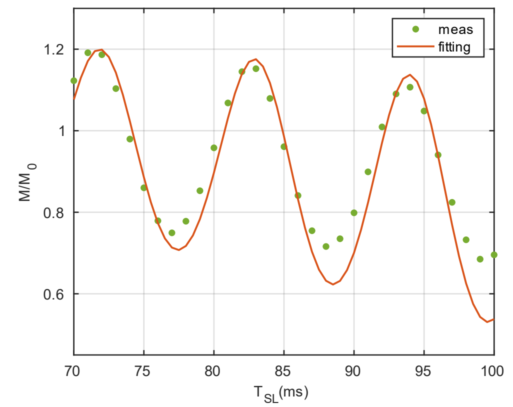

Supplementary figure 4: Measured BASL contrast as a function of the SL time and fitted curve.

5. SAR estimation

The specific absorption rate (SAR) limits the range of detectable frequencies for the SIRS approach, especially at high field strengths. Previous reports have calculated the SAR of the CRESL sequence; however, the values are highly dependent of sequence definition, coil used and subject scanned. Therefore, to assess the detection limits for our own experimental set-up, we used the SAR check computed by the scanner. In our case, we used a 64-channel head birdcage coil and set the subject values to typical values of high (170 cm) and weight (60 kg). The minimum T_R is limited by T_{SL}. Using the maximum allowed value, $T_{SL} = 100$ ms, we would have the highest temporal resolution and worst SAR case for the minimum possible $T_R = 165$ ms (minimum possible value given by preparation duration plus duration of the EPI readout). For this value, the obtained SAR as a function of F_{SL} is shown in Supplementary Figure 5.

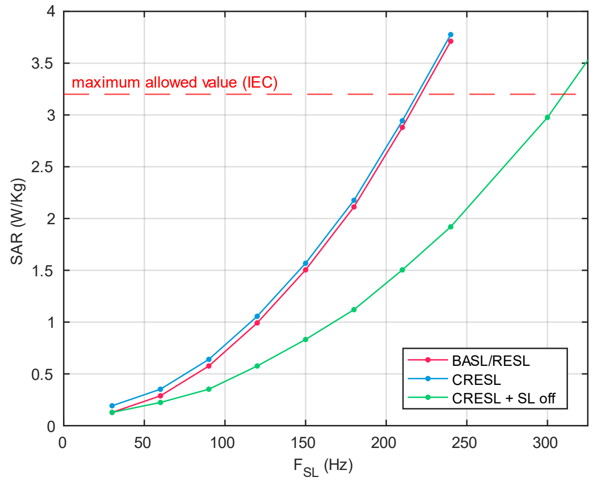

Supplementary figure 5. SAR estimation. Estimated SAR as a function of F_{SL} for both BASL and RESL, CRESL and the combined SL_{on}/SL_{off} approach. For all the sequences, $T_{SL} = 100$ ms, $T_R = 165$ ms, and the rest of the parameters are the same as described in part 2.3 of the Methods section.

The SAR value is the same for BASL and RESL, only differing with CRESL by the 180° pulse. The greatest contribution to SAR is given by the SL pulses, therefore it does not differ so much between the proposed preparations. In this setup, the frequency limit before exceeding the limit set by the International Electrotechnical Commission (IEC) (3.2 W/Kg for the head) is 222 Hz, 220 Hz and 310 Hz for BASL and CRESL and the SL_{on} (CRESL)/ SL_{off} approach respectively. If a higher frequency value must be used as target, T_R needs to be increased with the consequent loss of temporal resolution.

6. Measurement of individual signals

Supplementary Figure 6 shows the point-to-point division between the SL_{on} (CRESL) and SL_{off} acquisition of the 4 composite signals presented in Figure 7 a) of the main text. The signal shows traces of the 0.1 Hz oscillation, despite the acquisition being performed for F_{SL} equal to 30, 60 and 90 Hz. This influence is greater at 30 Hz and diminishes when increasing F_{SL}. This shows that slow drifts cannot be fully filtered out using the *ppd* between on and off acquisitions. The reason behind this is that the two acquisitions have a time difference of T_R. Therefore, any signal variation that occurs within a T_R will be registered differently in the SL_{on} and SL_{off} signal. An alternative to this is applying a high-pass filter to eliminate the influence of slow drifts if there is prior knowledge of the signals present. Nevertheless, the 90 Hz signal only has 10% of the amplitude of the slow-wave and is still the biggest detected component at $F_{SL} = 90$ Hz.

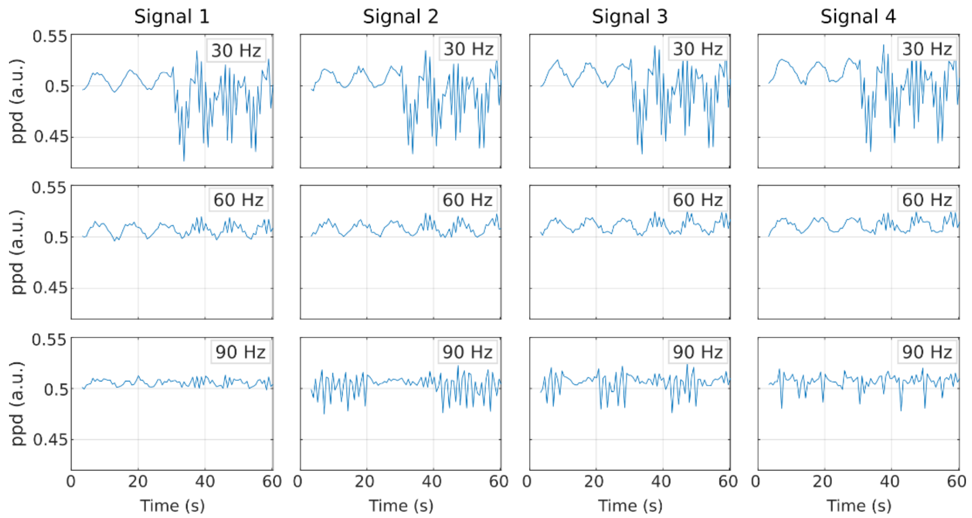

Supplementary figure 6: Point to point division (ppd) between SL_{on}(CRESL) and SL_{off} measured for the input signals shown in Figure 7 a). Each column corresponds to each of the input signals and each row to the ppd acquired at F_{SL} 30, 60 and 90 Hz.
